# Feasibility Study of MOS Gas Sensors for Detecting Mineral Hydrocarbon Contaminants in Freshly Harvested Olives at Different Maturity Stages

**DOI:** 10.3390/s26030816

**Published:** 2026-01-26

**Authors:** David Bonillo Martínez, Guilherme Felipe Pacheco Braga, Diego Manuel Martínez Gila, Silvia Satorres Martínez

**Affiliations:** 1 Robotics, Automation and Computer Vision Group, Electronic and Automation Engineering Department, University of Jaén, 23071 Jaén, Spainsatorres@ujaen.es (S.S.M.); 2 Institute for Olive Orchards and Olive Oils, University of Jaén, 23071 Jaén, Spain

**Keywords:** olive oil, MOSH, MOAH, gas sensors

## Abstract

The accidental contamination of olives by mineral hydrocarbons, such as diesel, motor lubricants, and hydraulic fluids from agricultural machinery, has become a growing concern in the olive oil industry. In response, European regulatory bodies are working on establishing new standards to address this issue. This study explores the feasibility of using Metal Oxide Semiconductor (MOS) gas sensors as a non-invasive method for detecting such contaminants on freshly harvested olives across different maturity stages. By assessing the sensitivity and selectivity of MOS sensors, this research aims to identify hydrocarbons that may adhere to the olive surface during harvesting and processing. The study involves controlled laboratory contamination scenarios, with samples exposed to various hydrocarbons to evaluate the relative response of individual MOS sensors under reproducible conditions. Findings from this research may provide valuable insights into rapid and cost-effective detection systems, supporting quality control and regulatory compliance in olive oil production, and contributing to the safety and traceability of olive-derived products. As a feasibility study, the results provide a basis for future developments involving multivariate analysis, field-contaminated samples, and industrial implementation.

## 1. Introduction

As the world’s largest producer of olive oil, Spain accounted for approximately 33% of global production in the 2023–2024 season, with an output of 854,000 tonnes, according to data published by the International Olive Council (IOC) [1]. This leadership has not only positioned the country as the leading exporter but has also had a significant impact on the international market. Due to its high nutritional value and distinctive flavor profile, olive oil is a fundamental component of the Mediterranean diet. Its consumption has increased significantly worldwide, with its quality standards regulated by the European Commission (EC), the IOC, and the Codex Alimentarius.

Given this scenario, a significant concern has emerged for the Spanish olive oil sector. Mineral oil hydrocarbons (MOH) comprise a complex mixture of isomers, categorized into MOSH (mineral oil saturated hydrocarbons) and MOAH (mineral oil aromatic hydrocarbons). Their presence in olive oils remains a pressing issue within the industry.

In 2023, the European Food Safety Authority (EFSA) updated its risk assessment on MOH in food. The assessment concluded that current dietary exposure to MOSH does not raise health concerns across all age groups. However, MOAH, particularly those with three or more aromatic rings, are associated with genotoxicity and carcinogenicity, posing potential health risks [2].

In response, the European Commission is advancing regulatory measures to establish maximum levels for MOAH in food. Draft amendments to Regulation (EU) 2023/915 propose specific limits for various food categories. For instance, olive oil is slated to have a maximum MOAH level of 2.0 mg/kg, while olive pomace oil is proposed to have a phased reduction: 10.0 mg/kg from 1 January 2026, decreasing to 5.0 mg/kg by 1 January 2026, and further to 2.0 mg/kg by 1 January 2026 [3].

These proposed regulations aim to enhance food safety and protect public health, underscoring the importance for producers to monitor and control MOH levels in olive oil products.

Once the problem has been identified, potential solutions are being explored to detect batches of olives contaminated with mineral oils and consequently with higher than recommended levels of MOSH and MOAH. Current methods for low quantification limits in the determination of these compounds are outlined in the ISO 20122:2024 Standard, utilizing online-coupled high performance liquid chromatography-gas and chromatography-flame ionization detection (HPLC-GC-FID) [4]. However, given the high cost and invasiveness of these traditional methods, researchers are investigating more economical strategies, albeit with reduced rigor and detection limits, to differentiate between contaminated and uncontaminated batches. This is especially important in the early stages of production, where early detection can prevent significant economic losses.

In this context, gas sensor–based approaches have already been explored for the detection of petroleum-derived hydrocarbon contamination in other matrices. For example, electronic nose systems based on MOS sensors have been successfully applied to detect and differentiate diesel and petrol contamination in soils, demonstrating the suitability of volatile-based sensing strategies for hydrocarbon detection in complex environments [5].

In an effort to discover novel detection methods, a feasibility study of gas sensors is proposed to detect contaminants in olives, specifically mineral oils and tractor fuel used during olive harvesting. Metal Oxide Semiconductor (MOS) gas sensors will be employed as a non-invasive and cost-effective method to determine their ability to differentiate between these contaminants and to identify the most suitable sensors for this application. Gas sensors have previously been used in various applications related to olive oil, such as identifying complex aromas in oils with the support of gas chromatography [6], differentiating and classifying olives based on geographical origin [7,8,9], and classifying olives [10,11] and olive oils [12,13,14] according to their quality parameters. This study constitutes a preliminary laboratory investigation under controlled conditions, designed to evaluate sensor sensitivity across a wide range of contamination levels. Future work will be directed toward validating these methods with field-collected olive samples. However, to the best of our knowledge, no previous studies have addressed the use of MOS sensors for detecting mineral hydrocarbon contaminants in olives, highlighting the novelty of this research and the need for feasibility assessment.

The following study will involve exposing the sensor array to small batches of olives with controlled levels of contamination, with the aim of determining whether gas sensors can detect contaminated olives and, if so, to what extent in terms of discrimination capability. The objective is not to quantify contamination levels or to establish calibration curves, but rather to evaluate the feasibility of individual MOS sensors to discriminate between contaminated and non-contaminated olive sample bags based on relative signal changes under controlled laboratory conditions. The sensors used will be from the MQ family, a type of MOS gas sensor equipped with an internal heater to ensure optimal operation. The resistance of these sensors varies upon detection of target gases and is measured using a voltage divider system based on this resistance value [15].

## 2. Materials and Methods

### 2.1. Sample Preparation

The experimental work carried out in this study spans two complete harvest seasons. A first campaign was conducted during the 2024–2025 season, and a second, fully replicated campaign was performed during the 2025–2026 season. Both campaigns followed exactly the same contamination and measurement protocol in order to evaluate the temporal reproducibility of the MOS sensor system under realistic agricultural variability. In each season, freshly harvested olives at two maturity stages (green and black) were collected and processed using identical preparation procedures, as detailed below.

Two complete experimental campaigns were carried out following exactly the same sample preparation protocol. Freshly harvested olives were collected from multiple olive trees located in the province of Jaén (Spain), all under comparable agronomic conditions. In each campaign, two maturity stages were evaluated: green olives and black olives. The first campaign used green olives harvested between October and November 2024 and black olives collected in January 2025. The second campaign replicated the same methodology using green olives harvested in early October 2025 and black olives collected in late November 2025. Olive maturity was not defined exclusively by harvest date, as ripening strongly depends on climatic conditions and significant variability can exist even within the same tree or orchard. Therefore, olives were selected based on maturity-related criteria rather than calendar date. Initial selection was performed using visual inspection and manual palpation, choosing fully black olives that were firm to the touch and not overripe. In addition, internal flesh coloration was visually assessed to ensure comparable maturity between campaigns.

Immediately after harvesting, the olives were transported to the laboratory and divided into two main groups: non-contaminated (ND) and intentionally contaminated (D). Contamination was performed using three petroleum-derived products commonly associated with harvesting machinery: John Deere Hy-Gard hydraulic oil (AcH), John Deere Plus-50 II engine oil (AcM), and diesel fuel (C) [16]. These products represent realistic contamination scenarios resulting from hydraulic leaks, engine oil residues, or diesel spills during collection operations.

**Figure 1:**
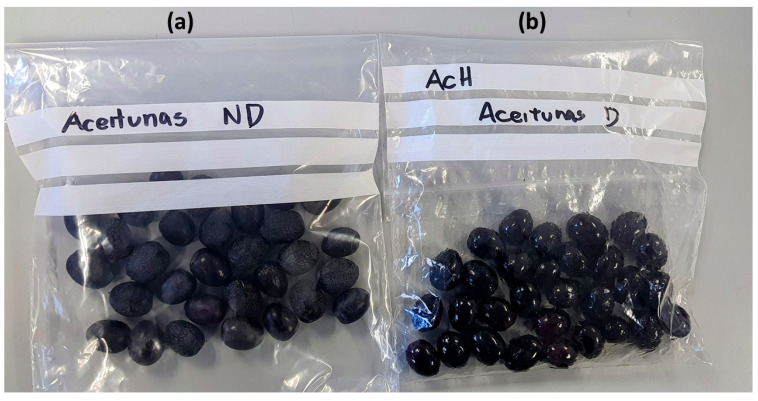
Black olives divided into two sample bags: (**a**) with 30 ND and (**b**) with 30 D olives, in this case, doped with AcH.

**Figure 2:**
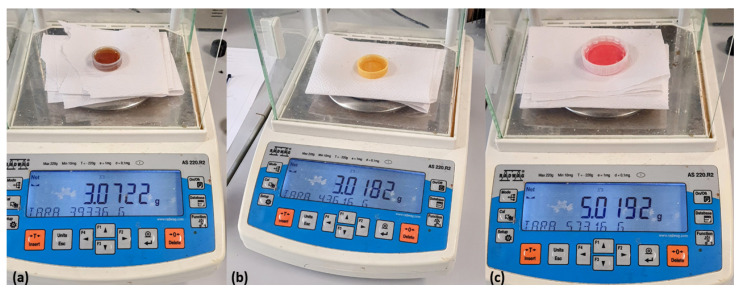
Weighing of contaminants, following the sequence: (**a**) AcM; (**b**) AcH; (**c**) C.

Figure 1 shows the initial division of olives into ND and D batches, each containing 30 olives. Contamination was performed separately for each petroleum-derived product. For each contaminant (AcM, AcH, and C), an independent D batch of 30 olives was prepared by adding a fixed amount of the corresponding product: two capfuls of AcM (6 g), two capfuls of AcH (6 g), or three capfuls of diesel fuel (15 g), as shown in Figure 2. These quantities were selected to emulate a high-exposure or worst-case contamination event, ensuring full impregnation of all fruits in the D batch. Although these levels exceed typical field contamination, they allow an initial feasibility evaluation of sensor sensitivity. Future work will calibrate contamination levels using samples obtained from real field incidents.

Due to manual handling during contamination, small variations in the applied contaminant mass were unavoidable. However, these variations were considered acceptable, as the objective of the study was to assess sensor sensitivity under high-exposure contamination scenarios rather than to perform quantitative dose–response analysis.

After contamination, the 30 ND and 30 D olives were mixed in predefined proportions to generate six contamination levels. As summarised in Table 1, sealed plastic containers, hereafter referred to as sample bags, were prepared, each containing ten olive samples with different ND/D ratios: 0%, 20%, 40%, 60%, 80% and 100% contamination. Three independent sample bags were prepared for each contamination level and for each contaminant, resulting in three replicates per level.

The same preparation procedure—including contamination quantities, ND/D mixing ratios, and number of replicates—was strictly followed in both campaigns to ensure full methodological consistency. After preparation, olive samples were placed in sealed sample bags and stored overnight at laboratory ambient temperature (approximately 25 °C). All samples were measured on the day following preparation using the acquisition protocol described in Section 2.3.

### 2.2. Experimental Setup

**Figure 3:**
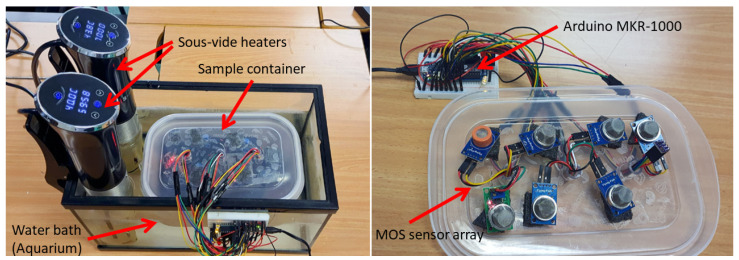
Real Experimental Setup.

**Figure 4:**
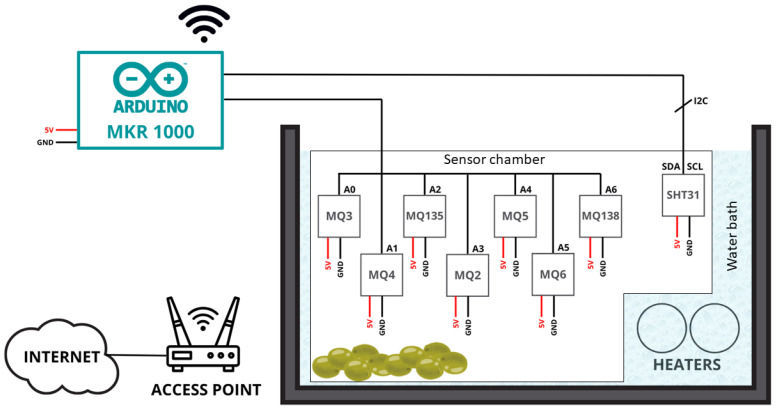
Schematic Experimental Setup.

As observed in Figure 3, a low-cost experimental setup was designed to achieve the study’s objectives. As shown in the schematic of Figure 4 the structure consists of an aquarium measuring 35 × 20 cm filled with water and equipped with two sous-vide immersion heaters (model KB552, Klarstein, Berlin, Germany), each with a nominal power of 1200 W, heaters to maintain a constant temperature. The aquarium was filled with approximately 3 L of water, providing a thermal buffer to ensure temperature stability during measurements. Water circulation was passively maintained by the operation of the sous-vide heaters, without additional mechanical stirring, as temperature gradients inside the bath were negligible under steady-state conditions.

Samples were placed in a tupperware container with holes drilled in the lid for sensor cables connecting to an Arduino MKR1000 (Arduino, Ivrea, Italy). The chosen MQ sensors are detailed in Table 2. They provide complementary sensitivity to a range of gases including alkanes, alkenes, alcohols, ammonia and aromatic compounds, as specified in their technical datasheets, ensuring broad coverage of volatile compounds potentially emitted by mineral oil contaminants. All MQ-series sensors used were sourced from Winsen Electronics Technology Co., Ltd. (Zhengzhou, China). The sensitive material for all sensors is tin dioxide ($SnO_{2}$), as specified in the manufacturer’s datasheets.

**Figure 5:**
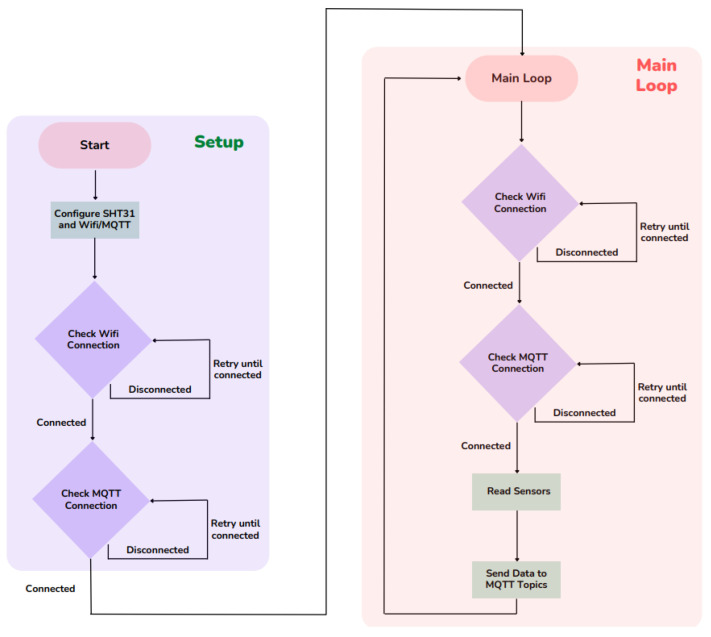
Arduino MKR1000 Flowchart.

The code compiled on the Arduino MKR1000 is based on two main groups: the structure of the setup and the main loop. The setup begins with the configuration of the SHT31 (Sensirion, Stafa, Switzerland) temperature and humidity sensor and the initialization of the connection with the WIFI and the MQTT (Message Queuing Telemetry Transport) connection. The WIFI configuration is then checked, and if it is disconnected, it remains in a loop until the connection is re-established. Once connected, the same procedure is performed, now with the MQTT connection. From this moment on, it moves to the Main Loop, in which both connections are checked again. Once the connections are confirmed, the sensor measurements are taken and the data is sent to the MQTT Topics. This process is repeated indefinitely as can be seen in the flowchart represented in Figure 5.

The MQTT messages are published to a Mosquitto broker hosted on a dedicated server maintained by the research group. On the same server, Telegraf is configured as a data collection agent that subscribes to the specified MQTT topics, parses the incoming sensor data, and forwards it to an InfluxDB time-series database. Each sensor was assigned to a dedicated MQTT topic, allowing structured data transmission.

These data acquisition and transmission technologies (MQTT, InfluxDB and Telegraf) are widely used in agricultural Internet of Things (IoT) systems and have been extensively described and evaluated in previous studies. Therefore, in the present work they are employed as a reliable support infrastructure for sensor data collection, without focusing on their technical implementation [17].

### 2.3. Measurement Procedure

Each measurement was performed using olive sample bags prepared at predefined contamination levels. At the beginning of a measurement cycle, the chamber was opened and one open sample bag was placed inside, with the olives facing upwards toward the sensor array. The chamber was then closed and the sample was exposed to the sensors for 25 min. During this period, sensor signals were continuously recorded. The heaters were set to 40 °C, resulting in an internal chamber temperature between 37 °C and 39 °C at the end of the exposure period.

Between consecutive measurements, the chamber and sensors were cleaned exclusively by forced air purging using a fan, with the chamber fully open. No solvents, detergents, or additional cleaning agents were used. This cleaning procedure was applied consistently between all measurements and was considered sufficient when sensor signals returned to baseline levels.

Two distinct stabilization periods were defined within each measurement cycle. The first stabilization period occurred immediately after the measurement step, with the chamber open, and lasted until sensor signals returned to a stable baseline. This stage ensured the removal of residual volatile compounds from the previous measurement.

After baseline recovery, the chamber was closed without any sample inside, and a second stabilization period was applied. This period was used to define the reference or “zero” point for subsequent measurements. Once stable baseline conditions were confirmed, the chamber was briefly opened to introduce the next sample bag, and the measurement phase started.

Although a minor transient temperature change occurred when opening the chamber to insert the sample bag, this effect was negligible compared to the total measurement duration and did not affect the stabilized sensor response used for analysis.

**Figure 6:**
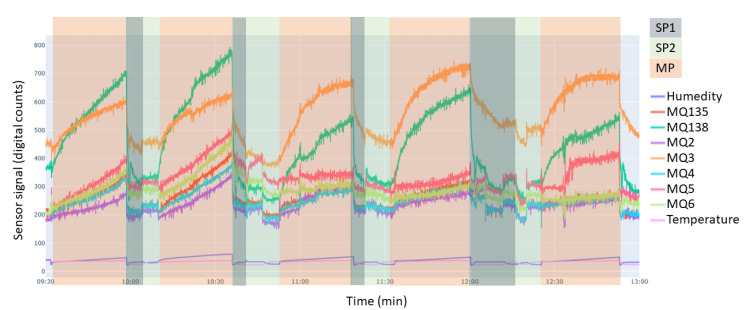
Representative time-series of MOS sensor signals recorded during consecutive measurement cycles. The raw digital output of each sensor (expressed as digital counts corresponding to the analog-to-digital converter output), together with temperature and humidity, is shown. Shaded regions indicate the main phases of the measurement cycle: sensor purging and baseline recovery (SP1), baseline stabilization with the chamber closed and no sample inside (SP2), and the sample measurement period (MP) after introducing the olive sample bag.

Sensor responses were recorded as time-series signals during the 25-min exposure period, as illustrated in Figure 6. For data analysis, a single scalar feature was extracted from each measurement. This feature was defined as the difference between the sensor signal at the end of the exposure period and the signal at the beginning of the exposure, after baseline stabilization. The resulting value represents a relative change in sensor signal, which may be positive or negative depending on the sensor type and its response direction.

For each contamination level, three independent measurements were performed. The complete measurement procedure was repeated independently for each contaminant and for both olive maturity stages. To minimize potential order effects caused by sensor saturation or memory, measurements were not performed sequentially from the lowest to the highest contamination level. Instead, the measurement order alternated between low and high contamination levels (e.g., 0–5–1–4–2–3), in order to mitigate possible carry-over or buffering effects in the sensors.

The sensors were not calibrated using certified gas mixtures, as the objective of this study was not quantitative gas concentration measurement, but rather to evaluate the feasibility of each individual MOS sensor to discriminate between contaminated and non-contaminated olive sample bags based on relative signal changes. All sensor responses were therefore expressed as relative changes with respect to a stabilized baseline, and sensor performance was evaluated using Analysis of Variance (ANOVA).

### 2.4. Data Analysis

To analyze the results obtained from the experiments, the statistical method of Analysis of Variance (ANOVA) will be employed. This method allows the study of separability between classes by defining a comparative variable and analyzing the difference between the means of the groups. Initially, a null hypothesis is defined in which it is considered that there is no significant difference between the means of the groups in question. The *p*-value is observed, which, if it is less than 0.05 for a single comparison, the null hypothesis is rejected, indicating a difference between the means of the groups and, consequently, the existence of separability between the classes. In this case, the degree of separability is determined by the F-Statistic, which defines the relationship between the variance between different groups and the variance within each group. A higher value of F indicates greater separability between these classes. In contrast, if the *p*-value exceeds 0.05, the null hypothesis is not rejected, indicating that there is no statistically significant difference between the means of the groups and, therefore, the inability to separate them.

For statistical analysis, samples were grouped into two classes. The Non-Contaminated (ND) class included only sample bags containing 10 ND olives (0% contamination), with three independent replicates. The Contaminated (D) class included all sample bags containing at least one contaminated olive (contamination levels 20% to 100%), resulting in a total of fifteen observations per contaminant and maturity stage. This grouping was intentionally selected to evaluate the binary discrimination capability of each sensor between contaminated and non-contaminated samples.

Although the analysis involves two classes, ANOVA was used for consistency across all sensors and campaigns. In the two-group case, ANOVA is equivalent to a *t*-test, with identical statistical conclusions.

## 3. Results

This section presents the results obtained from the two complete experimental campaigns carried out in this study. The first campaign (2024–2025 season) serves as the baseline evaluation of the MOS sensor array when exposed to olives contaminated with mineral hydrocarbons. The second campaign (2025–2026 season) replicates the full protocol using freshly harvested green and black olives from the new season. Presenting both campaigns separately allows the behaviour of the sensors to be evaluated under different natural conditions while maintaining identical experimental procedures, thus enabling an assessment of temporal reproducibility.

The results are organised in two main blocks: green olives and black olives. For each maturity stage, the behaviour of the sensors in the presence of each contaminant is analysed in order to determine which sensors respond best under each situation and how their performance varies between different ripening stages. This structure also facilitates a direct comparison of sensor behaviour between the two campaigns.

For each contaminant and maturity state, the results are first summarised in an ANOVA table reporting, for each sensor, the mean and standard deviation (Std Dev) of the extracted response (defined as the difference between the final reading and the initial value at the start of the measurement) for ND and D sample bags, together with the corresponding F-statistic and *p*-value. These tables allow a quantitative assessment of each sensor’s ability to discriminate between contaminated and non-contaminated samples.

Each ANOVA table is followed by a figure showing the corresponding sensor response curves. In these graphs, each point represents the mean amplitude of the extracted response for a given sensor and contamination level. This graphical representation facilitates a visual comparison of sensor responses across contamination levels.

Section 3.1 summarises the results obtained in the 2024–2025 season, while Section 3.2 reports the new findings corresponding to the 2025–2026 campaign.

### 3.1. Results—Campaign 2024–2025

#### 3.1.1. Green Olives

**Figure 7:**
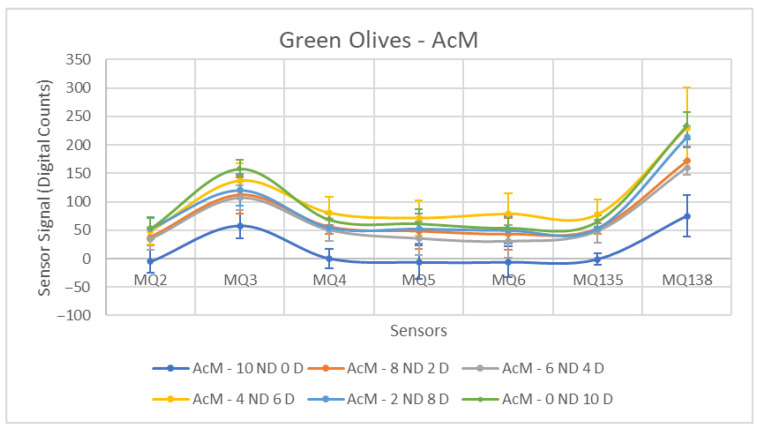
Comparative Analysis of AcM Contamination in Green Olives. Each point represents the mean value of the extracted sensor response obtained from three independent sample bags at each contamination level. Error bars indicate the dispersion between repetitions (standard deviation). Digital count corresponds to the sensor resistance value converted into a digital number through the analog-to-digital converter.

The first case to be analyzed concerns green olives contaminated with AcM. In Figure 7, a clear separation can be observed between the Non-Contaminated (10 ND, 0 D) and Contaminated olives groups. In the Contaminated olives, the formation of a group well separated from those that are Non-Contaminated is noted, which is reinforced when analyzing the ANOVA result presented in Table 3. It is interesting to observe that, in general, the behavior of the sensors is repeated for the different concentrations, making the represented curves very similar.

For all sensors, the *p*-value is less than 0.05, which allows us to reject the null hypothesis. Furthermore, we can perceive that the sensor that performs best in this situation is the MQ-135. This is due to the fact that it presents a low standard deviation for both groups compared to the other sensors, coupled with a high increase in the values measured by the sensors at the final instant, resulting in a high amplitude. With the values better grouped, there is greater repeatability of the measurements, allowing to identify the presence of contaminated olives in the respective samples. The opposite behavior is observed for the MQ-5 and MQ-6 sensors, which yields a relatively low F-Statistic value compared to the others. However, it is important to highlight that the jump between the means has greater relevance, given that in the MQ-138 sensor there is a high standard deviation for both groups, but it is compensated by the high difference between the means, since it has a good F-Statistic value compared to other sensors.

**Figure 8:**
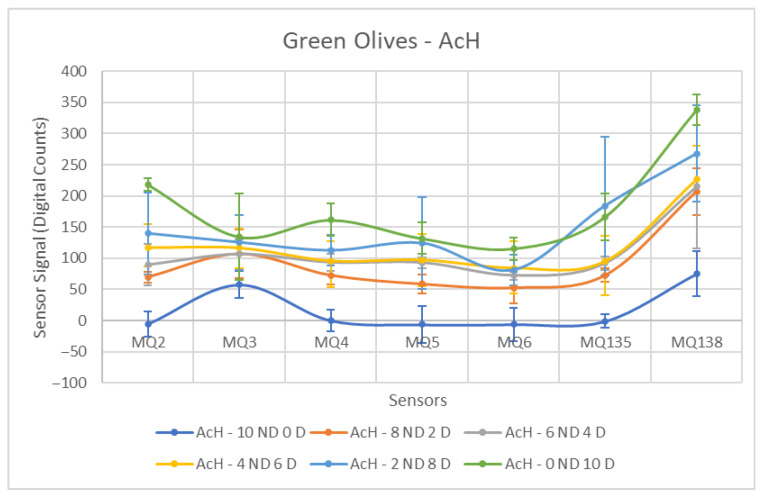
Comparative Analysis of AcH Contamination in Green Olives. Each point represents the mean value of the extracted sensor response obtained from three independent sample bags at each contamination level. Error bars indicate the dispersion between repetitions (standard deviation). Digital count corresponds to the sensor resistance value converted into a digital number through the analog-to-digital converter.

In the case of olives contaminated with hydraulic oil (AcH), a separation between both groups is generally feasible. As observed in Figure 8, the resulting curves do not exhibit the same stable behavior as in the previous scenario. Nevertheless, for most sensors, an incremental increase in the measured response can be observed as the proportion of contaminated olives increases. The sensor responses generally preserve the expected ordering from the lowest contamination level to the 100% contaminated samples. This behaviour suggests that, beyond binary discrimination between contaminated and non-contaminated samples, the sensor signals contain information related to contamination level, indicating potential feasibility for future multi-level classification.

As shown in Table 4, a significant standard deviation is observed in the Contaminated class, a consequence of the broadened range of values caused by increasing contaminant concentrations. Sensors exhibiting superior performance are those with more controlled standard deviations and a marked increase in the mean difference between the two groups, such as the MQ-4 and MQ-6. It is worth noting the contrasting behavior of the MQ-6, which was among the least effective in detecting engine oil (AcM) but demonstrates improved performance here, potentially due to the distinct composition of the contaminants and the volatile compounds present within them.

This behavioral shift is also observed in the MQ-135, which on this occasion exhibits a notably high standard deviation within the Contaminated group. This is accompanied by MQ-3, which also yields unfavorable results, mainly due to the lowest mean difference between groups among all sensors for this contaminant.

**Figure 9:**
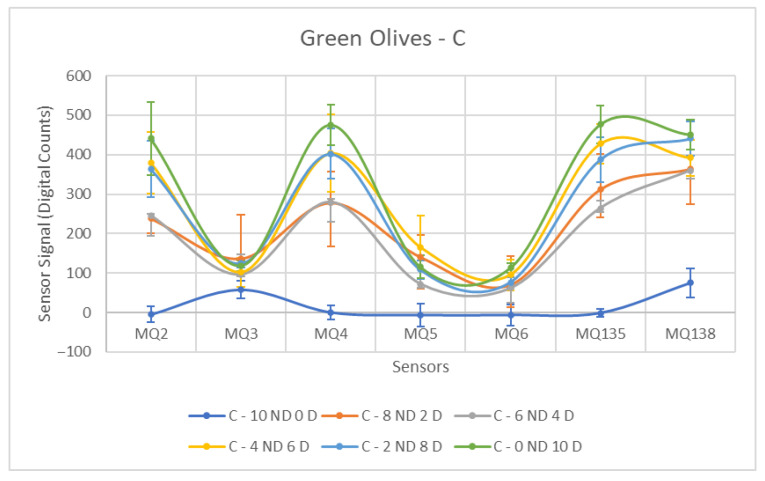
Comparative Analysis of C Contamination in Green Olives. Each point represents the mean value of the extracted sensor response obtained from three independent sample bags at each contamination level. Error bars indicate the dispersion between repetitions (standard deviation). Digital count corresponds to the sensor resistance value converted into a digital number through the analog-to-digital converter.

Diesel fuel (C) demonstrated the strongest sensor detection among the three contaminants, as shown in Table 5 and Figure 9. Its high volatile content resulted in a clear contrast between the groups, as evidenced by the significantly lower mean values in uncontaminated olives compared to contaminated ones. The presence of large standard deviations for select sensors within the Contaminated group is notable. These deviations are justified by substantial differences in the values obtained for varying contamination concentrations in the samples, where a jump in the measured value is observed with increasing contaminant concentration.

The statistical findings indicate that the F-Statistic values are generally consistently high, a result of the elevated mean values in the Contaminated class. In terms of sensor performance, the MQ-138 stands out, showing a significantly larger mean difference than other sensors for this contaminant and across all contaminants. Furthermore, the MQ-135 also displays a considerably high F-value in comparison to the remaining sensors.

When evaluating the F-values obtained for the sensors in samples contaminated with C, the MQ-2 and MQ-4 sensors can also be considered to exhibit adequate performance. Although they were not the most effective in identifying contaminated samples, their F-values, when compared to those obtained for other contaminants, were significantly elevated, representing a notable positive result.

#### 3.1.2. Black Olives

Firstly, the behavior of the sensors in response to black olives contaminated with AcM will be analyzed. A notable difference is observed compared to the previous results obtained for green olives, as summarized in Table 6. In this particular case, only a single sensor exhibits a *p*-value below 0.05. This indicates that for all other sensors, a separation between the Contaminated and Non-Contaminated olive groups is not possible. Furthermore, it is important to note that the F-statistic values are generally very low compared to those obtained for the olive tests in the earlier stage of maturity.

**Figure 10:**
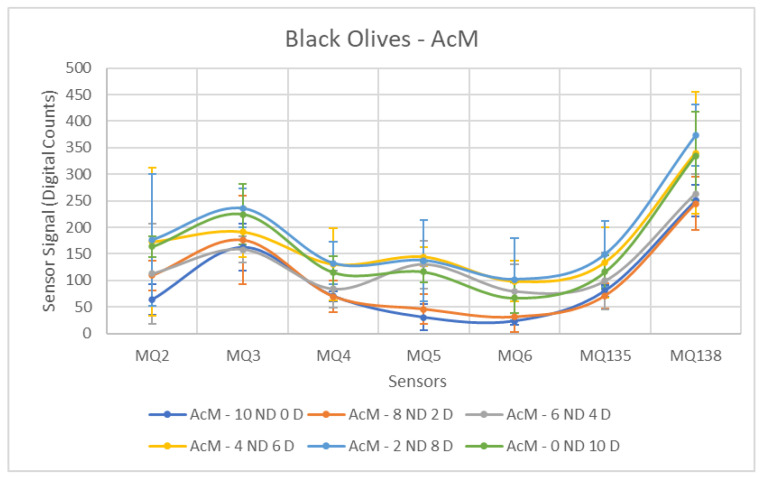
Comparative Analysis of AcM Contamination in Black Olives. Each point represents the mean value of the extracted sensor response obtained from three independent sample bags at each contamination level. Error bars indicate the dispersion between repetitions (standard deviation). Digital count corresponds to the sensor resistance value converted into a digital number through the analog-to-digital converter.

As depicted in Figure 10, a visual separation is discernible for most sensors from the 6 ND, 4 D concentration onward, compared to the Non-Contaminated sample. However, with the exception of the MQ-5 sensor, this difference is not pronounced, complicating the differentiation between classes and resulting in the previously mentioned *p*-values exceeding 0.05. For this specific sensor, MQ-5, separability is discernible without overlap between the Contaminated and Non-Contaminated groups. Furthermore, the MQ-6 sensor is of interest, showing a closely aligned behavior with a *p*-value relatively close to 0.05, indicating potential, but without conclusive distinction.

In contrast, the MQ-3 sensor displays the weakest performance. This is primarily caused by the elevated measurements in the Non-Contaminated samples, leading to an overlap of recorded values between the different groups.

**Figure 11:**
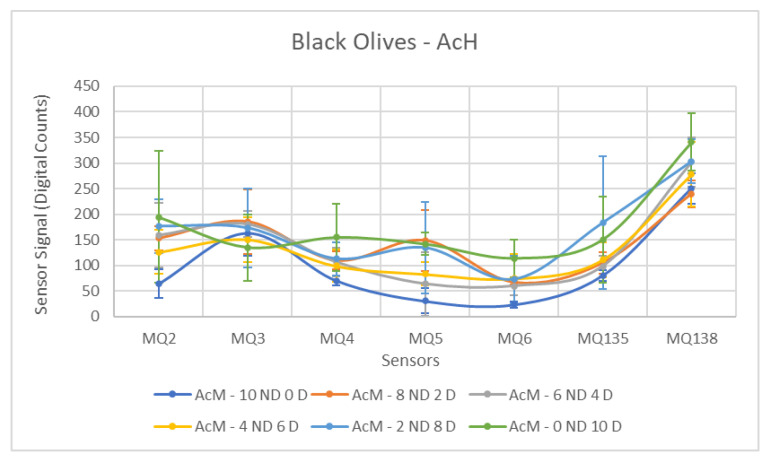
Comparative Analysis of AcH Contamination in Black Olives. Each point represents the mean value of the extracted sensor response obtained from three independent sample bags at each contamination level. Error bars indicate the dispersion between repetitions (standard deviation). Digital count corresponds to the sensor resistance value converted into a digital number through the analog-to-digital converter.

Subsequently, the values of the samples contaminated with AcH were examined. Similarly to the previous case, the sensors generally fail to differentiate the groups. This can be visually perceived by analyzing Figure 11, which clearly shows the overlap between the results of the Contaminated and Non-Contaminated samples, more pronounced than for the previous contaminant. It is interesting to note that the mean values for each concentration do not follow a pattern, appearing quite disordered and not respecting the order from the least to the most contaminated samples.

The sensors exhibiting the best performance were the MQ-2 and MQ-6, followed by the MQ-5. In these sensors, a *p*-value below the 0.05 threshold was observed, indicating separability between the groups. In general, the mean difference between the two groups was considerably smaller than that observed for other contaminants, resulting in the other half of the sensors not detecting this difference and producing statistical outcomes significantly below expectations.

Several sensors exhibited poor performance, notably MQ-3, MQ-135, and MQ-138. It is evident upon analyzing Table 7 that the mean values recorded for the different groups are very close, accompanied by elevated standard deviations due to disorder in the measurements of the Contaminated class, which resulted in unfavorable outcomes.

**Figure 12:**
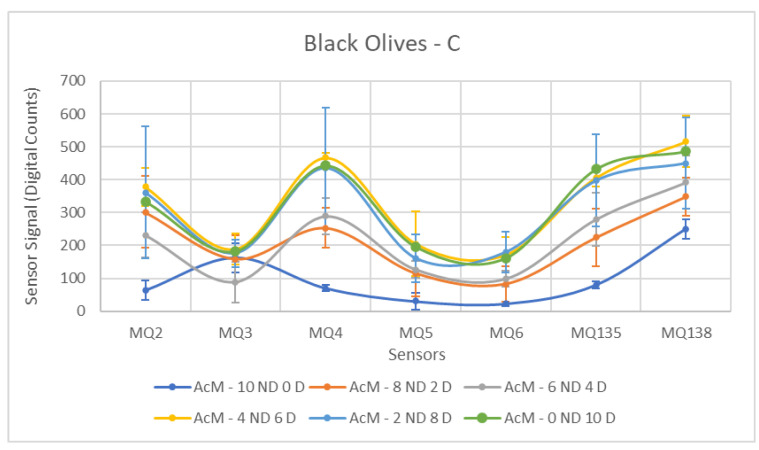
Comparative Analysis of C Contamination in Black Olives. Each point represents the mean value of the extracted sensor response obtained from three independent sample bags at each contamination level. Error bars indicate the dispersion between repetitions (standard deviation). Digital count corresponds to the sensor resistance value converted into a digital number through the analog-to-digital converter.

As expected, the results obtained for the samples contaminated with C produced the most favorable outcomes in terms of sensor detection, as summarized in Table 8. For all sensors, with the exception of MQ-3, the measured responses were satisfactory, allowing a clear differentiation between the two groups. Observing Figure 12, a pattern is discernible in the measurements, clearly indicating the separation between the Contaminated and Non-Contaminated samples.

Regarding the best performance sensors, MQ-2, MQ-4, and MQ-135 stand out, a result similar to the previous one for green olives. It is important to highlight that these sensors exhibit very high standard deviations in the Contaminated group, due to the significant jump observed with increasing contamination in the samples. However, by presenting significantly higher means than the Non-Contaminated group, their results are markedly superior to the other sensors.

Finally, the atypical behavior recorded by the MQ-3 sensor is worth noting. This sensor experienced considerable difficulties in the context of black olives, which becomes even more evident with this contaminant. Practically no evolution of the measured values is observed with increasing contamination levels, even reaching values lower than those of the Non-Contaminated samples.

### 3.2. Results—Campaign 2025–2026

#### 3.2.1. Green Olives

**Figure 13:**
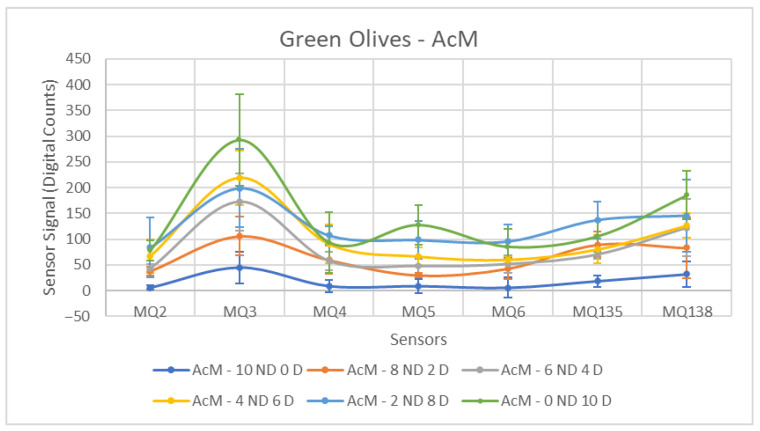
Comparative Analysis of AcM Contamination in Green Olives (2025–2026). Each point represents the mean value of the extracted sensor response obtained from three independent sample bags at each contamination level. Error bars indicate the dispersion between repetitions (standard deviation). Digital count corresponds to the sensor resistance value converted into a digital number through the analog-to-digital converter.

**Figure 14:**
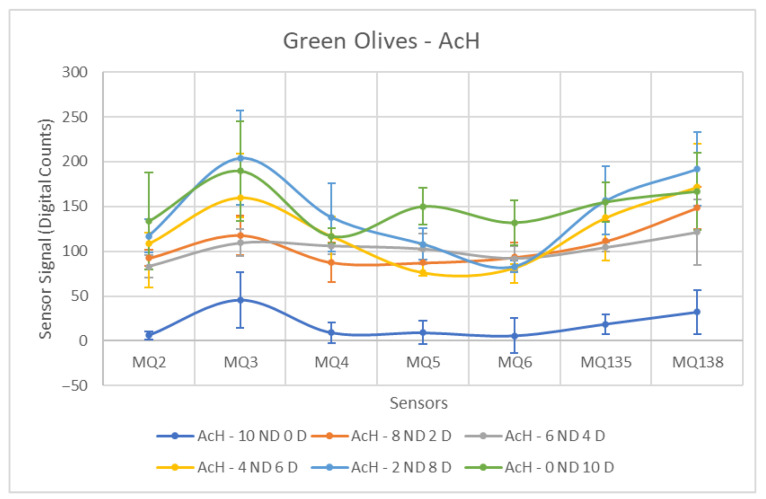
Comparative Analysis of AcH Contamination in Green Olives (2025–2026). Each point represents the mean value of the extracted sensor response obtained from three independent sample bags at each contamination level. Error bars indicate the dispersion between repetitions (standard deviation). Digital count corresponds to the sensor resistance value converted into a digital number through the analog-to-digital converter.

**Figure 15:**
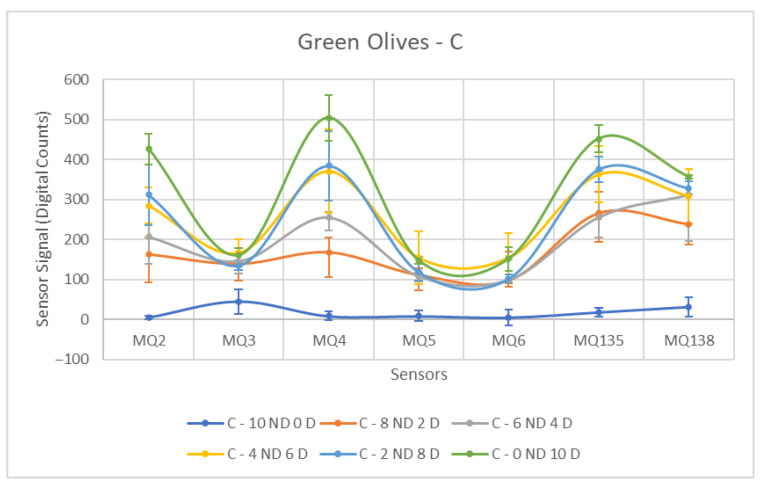
Comparative Analysis of C Contamination in Green Olives (2025–2026). Each point represents the mean value of the extracted sensor response obtained from three independent sample bags at each contamination level. Error bars indicate the dispersion between repetitions (standard deviation). Digital count corresponds to the sensor resistance value converted into a digital number through the analog-to-digital converter.

Figure 13, Figure 14 and Figure 15 show the response curves obtained for green olives contaminated with AcM, AcH and C in the 2025–2026 campaign. The characteristic shape observed in the previous season is preserved, with MQ135 and MQ138 consistently showing the highest signal amplitudes, and MQ5–MQ6 forming the lower segment of the sensor profile.

The ANOVA results for AcM contamination are shown in Table 9. All sensors exhibited statistically significant differences between ND and D samples. MQ135 presented the highest discrimination (F = 15.40), followed by MQ6 (F = 11.89) and MQ4 (F = 11.35). Although the separations were moderate compared to diesel, they were slightly stronger than those from the 2024–2025 campaign, suggesting a higher release of volatiles in this early-harvest season.

Table 10 summarises the ANOVA results for AcH. All sensors showed highly significant differences (*p* < 0.004). MQ4 exhibited the highest discrimination (F = 45.80), followed by MQ135 (F = 40.82) and MQ6 (F = 40.59). The magnitude of these values indicates that hydraulic oil generated a stronger and more distinct volatile profile in green olives during this season.

Diesel contamination produced the most pronounced differences among all contaminants, as shown in Table 11. All sensors achieved highly significant separations (*p* < 0.001), with MQ138 showing the highest F-statistic (F = 46.17), followed by MQ135 (F = 38.23) and MQ3 (F = 34.33). The large increase in amplitude after diesel contamination is consistent with the strong emission of volatile hydrocarbons associated with this product.

#### 3.2.2. Black Olives

**Figure 16:**
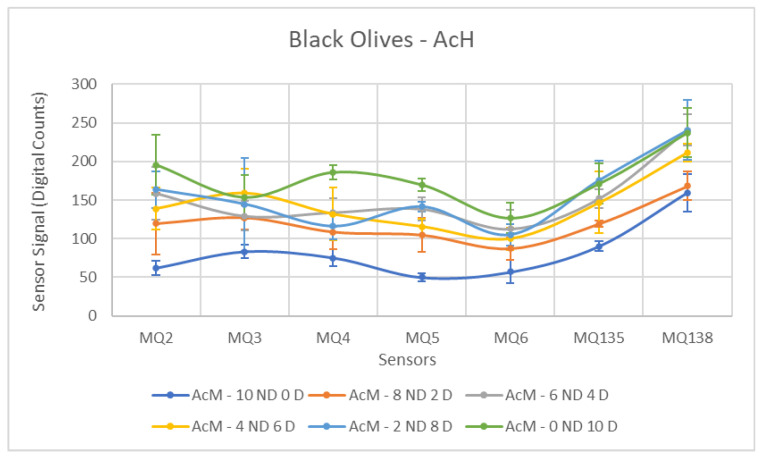
Comparative Analysis of AcH Contamination in Black Olives (2025–2026). Each point represents the mean value of the extracted sensor response obtained from three independent sample bags at each contamination level. Error bars indicate the dispersion between repetitions (standard deviation). Digital count corresponds to the sensor resistance value converted into a digital number through the analog-to-digital converter.

**Figure 17:**
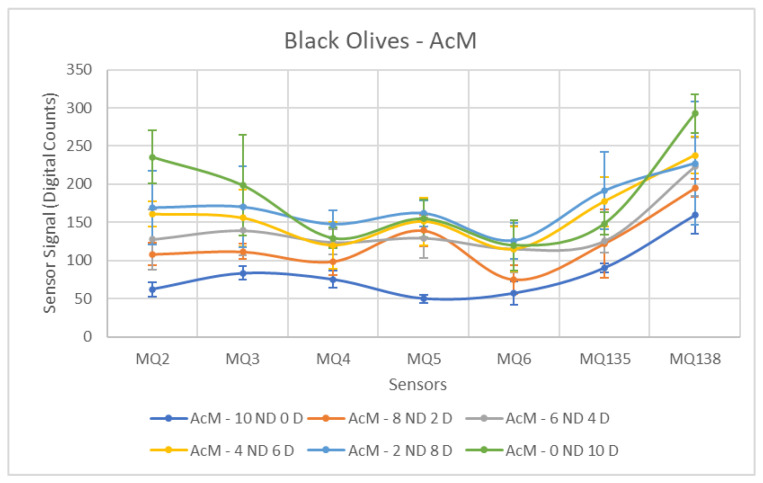
Comparative Analysis of AcM Contamination in Black Olives (2025–2026). Each point represents the mean value of the extracted sensor response obtained from three independent sample bags at each contamination level. Error bars indicate the dispersion between repetitions (standard deviation). Digital count corresponds to the sensor resistance value converted into a digital number through the analog-to-digital converter.

**Figure 18:**
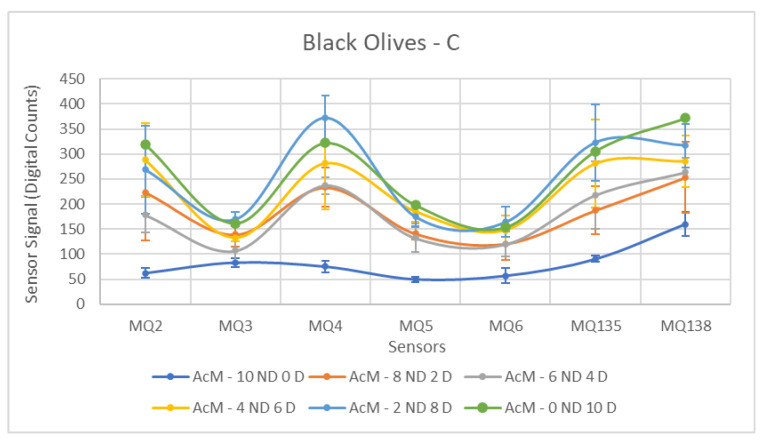
Comparative Analysis of C Contamination in Black Olives (2025–2026). Each point represents the mean value of the extracted sensor response obtained from three independent sample bags at each contamination level. Error bars indicate the dispersion between repetitions (standard deviation). Digital count corresponds to the sensor resistance value converted into a digital number through the analog-to-digital converter.

Figure 16, Figure 17 and Figure 18 show the sensor response curves for black olives contaminated with AcM, AcH and C. As in the previous campaign, black olives presented a richer baseline volatile profile, which reduced the contrast between ND and D samples for some contaminants. Nevertheless, significant separations were observed across all sensors.

Table 12 shows the ANOVA results for AcM contamination. All sensors exhibited significant differences between ND and D samples (*p* < 0.03). MQ5 showed the strongest discrimination (F = 47.11), in agreement with the behaviour observed in the previous season. MQ4 (F = 11.80) and MQ2 (F = 9.63) also contributed notably to the separation. However, the overall contrast remained lower than that obtained in green olives.

The ANOVA results for AcH, shown in Table 13, indicate that hydraulic oil produced moderate but consistent separations across all sensors. MQ5 (F = 29.51) and MQ2 (F = 16.52) were again the most discriminative sensors. These results confirm the difficulty of detecting AcH in fully ripe olives, despite the statistically significant differences observed.

Diesel contamination again produced highly distinguishable responses in black olives (Table 14). MQ5 and MQ4 were the most discriminative sensors (F = 33.40 and 25.54, respectively), followed by MQ2 (F = 17.39). MQ135 and MQ138 also showed significant discrimination. These results corroborate diesel as the contaminant that is most easily detected by the sensor array, even in fruit with a more complex endogenous volatile composition.

## 4. Discussion

The two experimental campaigns conducted across consecutive harvest seasons provide a consistent body of evidence supporting the feasibility of MOS gas sensors for detecting mineral hydrocarbon contaminants in freshly harvested olives. Despite natural seasonal variability in fruit composition, the global behaviour of the sensor array remained stable, and the characteristic sensitivity patterns of the MQ-series sensors were preserved throughout all measurements.

A first relevant observation is the strong influence of olive maturity on the detectability of contaminants. Green olives, characterised by a lower endogenous volatile load, produced higher separability between non-contaminated and contaminated samples for all contaminants. In contrast, black olives exhibited greater baseline complexity due to their advanced ripening stage, which attenuated the relative impact of AcM and AcH contamination and reduced separability for some sensors. These findings are consistent with previous studies reporting higher concentrations of alcohols, aldehydes and light hydrocarbons in ripe olives, which can interfere with MOS sensor responses.

Across all experiments, diesel was the contaminant that consistently generated the strongest and most easily detectable signature. This behaviour is expected due to the high volatility of diesel and has been similarly reported in studies using MOS sensors for petroleum-derived compounds. AcH and AcM also produced significant changes in sensor response, but with lower magnitude, reflecting their more limited volatile fraction. These differences are particularly important from a practical standpoint, as diesel leaks are among the most common contamination sources in harvesting operations.

Another key aspect is the reproducibility observed between the two campaigns. The ranking of relative sensor responses (MQ135 and MQ138 > MQ4 and MQ2 > MQ5 and MQ6) was identical across years, and the magnitude and direction of the class separations were preserved. This reproducibility supports the robustness of this sensing strategy and suggests that MOS sensors can provide reliable performance even when applied to different harvest seasons or batches.

Nevertheless, several limitations must be acknowledged. Although blank samples and stabilisation routines were used to minimise short-term drift, long-term drift was not assessed, as measurements were performed within short controlled sessions. In addition, only one physical unit of each sensor type was used, meaning that inter-unit variability—known to be relevant in MOS sensors—was not evaluated. Furthermore, while environmental conditions were stabilised during the experiments, no systematic characterisation of humidity cross-sensitivity was carried out, despite the recognised influence of humidity on MOS sensor performance.

Finally, the analysis presented in this study relies on single-sensor statistical separability through ANOVA. While this approach effectively demonstrates feasibility, future work should incorporate multivariate techniques such as Principal Component Analysis (PCA), Linear Discriminant Analysis (LDA), or machine learning classifiers, including neural networks. Such methodologies may significantly enhance discrimination, particularly in complex scenarios such as ripe olives or low contamination levels, and will be essential for the development of a fully functional electronic nose system adapted to industrial environments.

Overall, the results obtained demonstrate that low-cost MOS gas sensors constitute a promising tool for the rapid, non-invasive detection of mineral hydrocarbon contamination in olives. Their reproducibility across seasons, sensitivity to multiple contaminant types, and low hardware cost make them strong candidates for integration into early-stage quality control workflows in olive oil production.

## 5. Conclusions

This study demonstrated the feasibility of using low-cost MOS gas sensors as a non-invasive approach for detecting mineral hydrocarbon contamination (MOSH/MOAH-related substances) in freshly harvested olives. Two complete experimental campaigns conducted in consecutive harvest seasons confirmed the reproducibility and robustness of the sensing approach under realistic agronomic variability.

Across both campaigns, the sensor array consistently detected significant differences between non-contaminated and contaminated olive samples. The best-performing sensors in green olives were MQ135 for AcM, MQ4 and MQ6 for AcH, and MQ138 and MQ135 for diesel. In black olives, where endogenous volatile compounds are naturally more abundant—particularly alcohols, aldehydes and light hydrocarbons [18]—the relative contrast between contaminated and non-contaminated samples decreased. Even so, clear separability was achieved, with MQ5 and MQ6 showing the best performance for AcM, MQ4 and MQ2 for AcH, and MQ2, MQ4 and MQ135 for diesel. The elevated baseline in ripe olives also explains the reduced efficacy of sensors such as MQ3 and MQ138, whose sensitivity overlaps with naturally emitted volatiles in this advanced maturation stage.

The results indicate that MOS sensors can be a promising tool for early-stage quality control in olive oil production, enabling rapid screening of contaminated batches and supporting compliance with increasingly stringent regulations on mineral hydrocarbons. The simplicity, low cost and non-invasive nature of the proposed system make it suitable for future implementation at olive mill intake points.

This study was conducted under controlled laboratory conditions using high-exposure contamination scenarios and a limited number of replicates. Future work should focus on validation with real field-contaminated olives, evaluation of long-term drift and environmental cross-sensitivities, and the integration of multivariate analysis or machine learning techniques to enhance discrimination in complex scenarios, particularly for ripe olives.

## Figures and Tables

**Table 1:** Sample composition according to contamination level (used in both campaigns).

Contamination Levels in Sample Bags	Sample Bags Composition
0	10 ND, 0 D
1	8 ND, 2 D
2	6 ND, 4 D
3	4 ND, 6 D
4	2 ND, 8 D
5	0 ND, 10 D

**Table 2:** Sensor Definition and Sensitivities.

Reference	Sensor Name	High Sensitivity
1	MQ-2	Liquefied Petroleum Gas (LPG), Propane, Hydrogen
2	MQ-3	Alcohol Gas, Benzene
3	MQ-4	Natural Gas, Methane
4	MQ-5	LPG, Natural Gas, Town Gas
5	MQ-6	Combustible Gas, Propane, Butane, LPG
6	MQ-135	Ammonia Gas, Sulfide, Nitrogen Oxides, Benzene, Smoke
7	MQ-138	Hexane, Benzene, Ammonia Gas, Alcohol, Smoke

**Table 3:** ANOVA Analysis for Sensors Response to Green Olives with AcM. Response is defined as the difference between the sensor signal at the end of the exposure period and the initial stabilized baseline value.

Sensor	Mean (ND)	Std Dev (ND)	Mean (D)	Std Dev (D)	F-Statistic	*p*-Value
MQ2	−5.33	20.01	45.47	18.26	18.88	0.000502
MQ3	57.67	22.14	126.87	29.55	14.50	0.001545
MQ4	0.33	17.39	61.93	18.47	28.21	0.00007
MQ5	−6.33	29.26	53.87	27.66	11.67	0.00354
MQ6	−6.00	26.91	51.20	29.36	9.68	0.00671
MQ135	−1.00	10.39	59.53	17.42	32.82	0.00003
MQ138	75.00	35.93	201.93	44.33	21.42	0.00028

**Table 4:** ANOVA Analysis for Sensors Response to Green Olives with AcH. Response is defined as the difference between the sensor signal at the end of the exposure period and the initial stabilized baseline value.

Sensor	Mean (ND)	Std Dev (ND)	Mean (D)	Std Dev (D)	F-Statistic	*p*-Value
MQ2	−5.33	20.01	126.80	62.07	12.76	0.0025
MQ3	57.67	22.14	117.93	38.96	6.53	0.0211
MQ4	0.33	17.39	107.00	36.78	23.29	0.0002
MQ5	−6.33	29.26	100.87	43.22	16.50	0.0009
MQ6	−6.00	26.91	81.07	30.44	21.02	0.0003
MQ135	−1.00	10.39	121.93	66.05	9.86	0.0063
MQ138	75.00	35.93	250.80	73.74	15.71	0.0011

**Table 5:** ANOVA Analysis for Sensors Response to Green Olives with C. Response is defined as the difference between the sensor signal at the end of the exposure period and the initial stabilized baseline value.

Sensor	Mean (ND)	Std Dev (ND)	Mean (D)	Std Dev (D)	F-Statistic	*p*-Value
MQ2	−5.33	20.01	333.60	100.53	32.29	0.0000396
MQ3	57.67	22.14	115.47	21.97	17.26	0.0007454
MQ4	0.33	17.39	367.87	104.35	35.30	0.0000207
MQ5	−6.33	29.26	120.47	55.52	14.33	0.0016199
MQ6	−6.00	26.91	83.40	35.65	16.61	0.0008799
MQ135	−1.00	10.39	373.60	90.70	48.65	0.00000313
MQ138	75.00	35.93	401.27	59.39	81.94	0.00000011

**Table 6:** ANOVA Analysis for Sensors Response to Black Olives with AcM. Response is defined as the difference between the sensor signal at the end of the exposure period and the initial stabilized baseline value.

Sensor	Mean (ND)	Std Dev (ND)	Mean (D)	Std Dev (D)	F-Statistic	*p*-Value
MQ2	64.00	28.58	146.93	85.72	2.63	0.1242
MQ3	162.67	43.88	197.20	54.44	1.05	0.3203
MQ4	70.33	9.71	106.47	44.99	1.83	0.1949
MQ5	30.33	24.50	115.07	52.09	7.33	0.0156
MQ6	23.33	6.66	75.87	48.75	3.31	0.0877
MQ135	80.33	11.06	113.93	51.23	1.22	0.2855
MQ138	250.33	29.57	310.93	80.53	1.59	0.2258

**Table 7:** ANOVA Analysis for Sensors Response to Black Olives with AcH. Response is defined as the difference between the sensor signal at the end of the exposure period and the initial stabilized baseline value.

Sensor	Mean (ND)	Std Dev (ND)	Mean (D)	Std Dev (D)	F-Statistic	*p*-Value
MQ2	64.00	28.58	161.80	65.18	6.26	0.0236
MQ3	162.67	43.88	165.07	52.79	0.01	0.9425
MQ4	70.33	9.71	116.53	38.66	4.04	0.0615
MQ5	30.33	24.50	114.33	60.03	5.46	0.0327
MQ6	23.33	6.66	77.47	35.52	6.60	0.0206
MQ135	80.33	11.06	129.67	69.51	1.43	0.2486
MQ138	250.33	29.57	292.80	53.96	1.70	0.2111

**Table 8:** ANOVA Analysis for Sensors Response to Black Olives with C. Response is defined as the difference between the sensor signal at the end of the exposure period and the initial stabilized baseline value.

Sensor	Mean (ND)	Std Dev (ND)	Mean (D)	Std Dev (D)	F-Statistic	*p*-Value
MQ2	64.00	28.58	321.00	107.51	16.16	0.000989
MQ3	162.67	43.88	158.93	57.32	0.01	0.917091
MQ4	70.33	9.71	377.53	127.29	16.63	0.000876
MQ5	30.33	24.50	160.60	72.61	9.05	0.008338
MQ6	23.33	6.66	138.67	56.72	11.79	0.003411
MQ135	80.33	11.06	347.87	111.66	16.38	0.000935
MQ138	250.33	29.57	438.93	95.41	11.01	0.004345

**Table 9:** ANOVA results for green olives contaminated with engine oil (AcM), 2025–2026 campaign. Response is defined as the difference between the sensor signal at the end of the exposure period and the initial stabilized baseline value.

Sensor	Mean (ND)	Std Dev (ND)	Mean (D)	Std Dev (D)	F-Statistic	*p*-Value
MQ2	6.00	4.58	62.20	32.58	8.48	0.0102
MQ3	45.33	30.92	197.80	83.52	9.34	0.007545
MQ4	9.00	11.36	81.73	36.23	11.35	0.003903
MQ5	9.00	13.00	74.20	42.21	6.73	0.019598
MQ6	5.67	19.60	67.27	29.28	11.89	0.00331
MQ135	18.67	11.15	96.67	33.33	15.40	0.001209
MQ138	32.00	24.43	132.67	56.79	8.75	0.009265

**Table 10:** ANOVA results for green olives contaminated with hydraulic oil (AcH), 2025–2026 campaign. Response is defined as the difference between the sensor signal at the end of the exposure period and the initial stabilized baseline value.

Sensor	Mean (ND)	Std Dev (ND)	Mean (D)	Std Dev (D)	F-Statistic	*p*-Value
MQ2	6.00	4.58	106.80	29.34	33.60	0.000027
MQ3	45.33	30.92	156.20	52.83	11.99	0.003202
MQ4	9.00	11.36	113.13	25.65	45.80	0.000005
MQ5	9.00	13.00	104.87	31.50	25.83	0.000111
MQ6	5.67	19.60	96.40	22.90	40.59	0.000009
MQ135	18.67	11.15	133.00	29.95	40.82	0.000009
MQ138	32.00	24.43	160.07	41.39	26.06	0.000106

**Table 11:** ANOVA results for green olives contaminated with diesel (C), 2025–2026 campaign. Response is defined as the difference between the sensor signal at the end of the exposure period and the initial stabilized baseline value.

Sensor	Mean (ND)	Std Dev (ND)	Mean (D)	Std Dev (D)	F-Statistic	*p*-Value
MQ2	6.00	4.58	278.87	107.33	18.46	0.000554
MQ3	45.33	30.92	149.60	27.72	34.33	0.000024
MQ4	9.00	11.36	336.60	134.09	17.04	0.00079
MQ5	9.00	13.00	128.07	36.20	30.34	0.000048
MQ6	5.67	19.60	121.13	38.24	25.11	0.000128
MQ135	18.67	11.15	342.67	88.47	38.23	0.000013
MQ138	32.00	24.43	308.73	68.22	46.17	0.000004

**Table 12:** ANOVA results for black olives contaminated with engine oil (AcM), 2025–2026 campaign. Response is defined as the difference between the sensor signal at the end of the exposure period and the initial stabilized baseline value.

Sensor	Mean (ND)	Std Dev (ND)	Mean (D)	Std Dev (D)	F-Statistic	*p*-Value
MQ2	62.33	9.29	160.27	53.23	9.63	0.006837
MQ3	83.33	9.02	155.07	47.92	6.37	0.022555
MQ4	75.33	11.15	123.93	23.54	11.80	0.00340
MQ5	50.00	5.29	147.27	23.87	47.11	0.000004
MQ6	57.00	15.13	110.33	29.78	8.84	0.008968
MQ135	90.33	6.11	153.00	40.94	6.67	0.020003
MQ138	159.67	24.17	235.00	49.30	6.45	0.021864

**Table 13:** ANOVA results for black olives contaminated with hydraulic oil (AcH), 2025–2026 campaign. Response is defined as the difference between the sensor signal at the end of the exposure period and the initial stabilized baseline value.

Sensor	Mean (ND)	Std Dev (ND)	Mean (D)	Std Dev (D)	F-Statistic	*p*-Value
MQ2	62.33	9.29	155.33	38.52	16.52	0.000902
MQ3	83.33	9.02	142.80	32.26	9.60	0.006904
MQ4	75.33	11.15	135.27	33.64	8.93	0.008689
MQ5	50.00	5.29	133.93	26.04	29.51	0.000055
MQ6	57.00	15.13	106.27	20.43	15.41	0.001206
MQ135	90.33	6.11	153.00	29.46	12.85	0.002478
MQ138	159.67	24.17	219.73	36.39	7.32	0.01558

**Table 14:** ANOVA results for black olives contaminated with diesel (C), 2025–2026 campaign. Response is defined as the difference between the sensor signal at the end of the exposure period and the initial stabilized baseline value.

Sensor	Mean (ND)	Std Dev (ND)	Mean (D)	Std Dev (D)	F-Statistic	*p*-Value
MQ2	62.33	9.29	255.93	78.39	17.39	0.000722
MQ3	83.33	9.02	142.07	34.62	8.14	0.011492
MQ4	75.33	11.15	289.13	71.38	25.54	0.000117
MQ5	50.00	5.29	166.40	33.98	33.40	0.000028
MQ6	57.00	15.13	141.33	34.20	16.90	0.000818
MQ135	90.33	6.11	263.07	77.71	14.10	0.001727
MQ138	159.67	24.17	298.20	62.45	13.77	0.001901

## Data Availability

The data presented in this study are available from the corresponding author upon reasonable request.
